# AIT (allergen immunotherapy): a model for the “precision medicine”

**DOI:** 10.1186/s12948-015-0028-6

**Published:** 2015-10-08

**Authors:** Giovanni Passalacqua, Giorgio Walter Canonica

**Affiliations:** Allergy and Respiratory Diseases, Department of Internal Medicine, S. Martino Hospital, IRCCS San Martino-IST-University of Genoa, Pad.Maragliano, L.go R Benzi 10, 16133 Genoa, Italy

**Keywords:** Precision medicine, Personalized medicine, Allergen immunotherapy

## Abstract

The interpretation of medical approaches, especially therapy, evolved rapidly in the last century. Starting from the simple description of symptoms, we moved to the pathophysiological descriptions, to the evidence-based medicine, until the so-called “precision medicine”. This latter can be defined as a structural model aimed at customizing healthcare, with medical decisions/products tailored on an individual patient at a highly detailed level. In this sense, allergen immunotherapy represents an optimal model of “precision medicine”, since we know and describe symptoms, function, aetiological agents at molecular level, and we have the possibility to intervene on the natural history of the disease. If considered under the point of view of pharmaco-economy, that is prescribing the optimal treatment to the right patient, allergen immunotherapy represents an almost-ideal model of precision medicine.

## General aspects

The medical science slowly evolved, along centuries, from the Hippocratic “humours” [1] to a more pathophysiology-oriented interpretation of clinical phenomena [2–4], until the current “omic” sciences. Thus, it seems that a more and more potent magnifying lens has become available to study and understand diseases. In addition, it is clear that the clinical science, the mechanistic knowledge and the translational applications are becoming more and more strictly interconnected.

From the clinical point of view (essentially the therapeutic aspect), a “blockbuster” approach was applied in clinical practice during the last decades. This attitude was likely due to a superficial and incomplete knowledge of the underlying mechanisms of diseases. The advanced insights on the mechanisms and the specific features of patients’ groups leaded to the definition of phenotypes and endotypes [5, 6]. This “stratification” of groups, in turn, provided a more detailed definition of diagnosis and treatments, consequently resulting in more appropriate definition of the eligibility of patients to the different therapeutic tools. The “phenotype driven therapy” is now a real and urging need, especially when expensive drugs, such as biologicals/biosimilars have to be prescribed [7, 8]. This aspect is unavoidably linked to sustainability for HealthCare Systems, which will afford relevant economic burdens. These latter will grow dramatically targeting, for instance, 30 % of Gross Incoming Product in 2040 in the US [9]. According to those premises we have to face two major reasons for a more selective approach (phenotyping/endotyping) to the patients who are potentially candidates to an effective treatment:

- medical/scientific aspects
- pharmaco-economic aspects.

The “blockbuster approach” (i.e. one size fits all) cannot be currently used with many of the very expensive treatments available, where the best cost/effective treatment should be provided. This also implies a greater professional and responsible involvement of specialists in properly selecting patients. The “precision medicine” can be defined as a structural model aimed at customizing healthcare at best, with medical decisions, practices, and/or products tailored on an individual patient. The term of “personalized medicine” is also used interchangeably.

The major goals of “personalized medicine” are essentially:

- to improve the clinical outcomes and their predictability;
- to reduce the side effects caused by a possibly inappropriate treatment;
- to increase the quality of life;
- to encourage patients’ compliance due to a perceived clinical improvement;
- to optimise the use of healthcare resources.

**Fig. 1:**
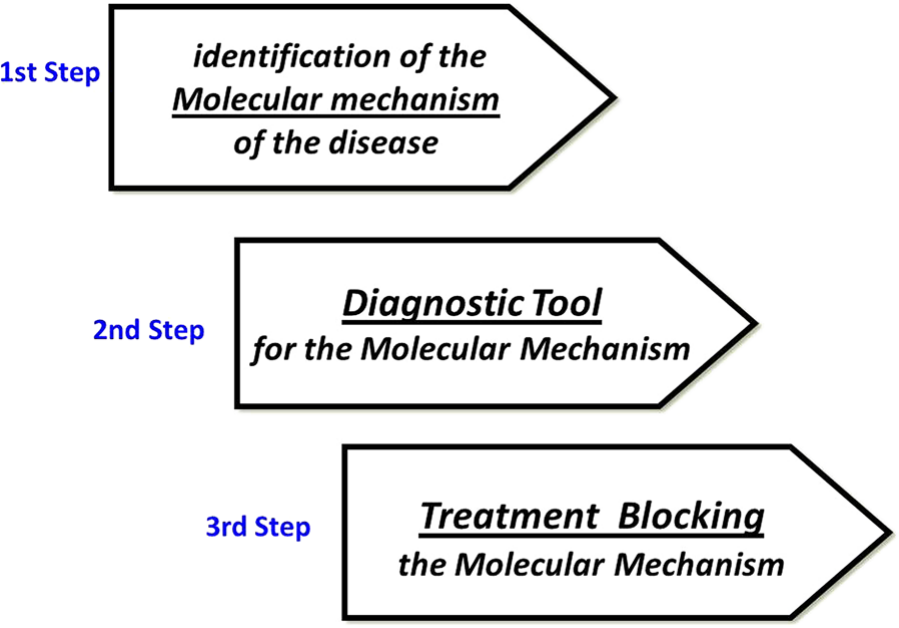
The consequential process underlying personalized medicine

Hamburg and Collins [10], described the path to a personalized medicine as summarized in Fig. 1, highlighting the relevant economic investments to pursue this approach: cost/effective medicine. A pertinent example is the identification, in cystic fibrosis, of one of the molecular mechanisms that cause the disease: this is present in only 4 % of patients, but once they are identified, an effective treatment, although expensive, can be given with expected positive results [11]. Another explanatory example is the specific antagonism to IL-5 in eosinophilic-driven asthma. Also in this case, it is possible to reasonably identify a priori those candidate patients who will respond to the targeted biological treatment [12, 13].

Personalized medicine is still a critical aspect in the most complex, prevalent and expensive chronic diseases, such as COPD [14] or asthma in general [15], where a targeted approach would heavily affect the management.

## Allergen immunotherapy (AIT)

Allergen immunotherapy remains one of the best candidates to a personalized-medicine approach since we currently know: (a) the main immunological and molecular events underlying the allergic symptoms [16]; (b) which are the more specific and sensitive standard diagnostic tests to identify the IgE-mediated reactions; (c) the relevant molecules involved in allergic reactions [17, 18]; (d) purified and standardized documented products for effective and safe AIT.

**Fig. 2:**
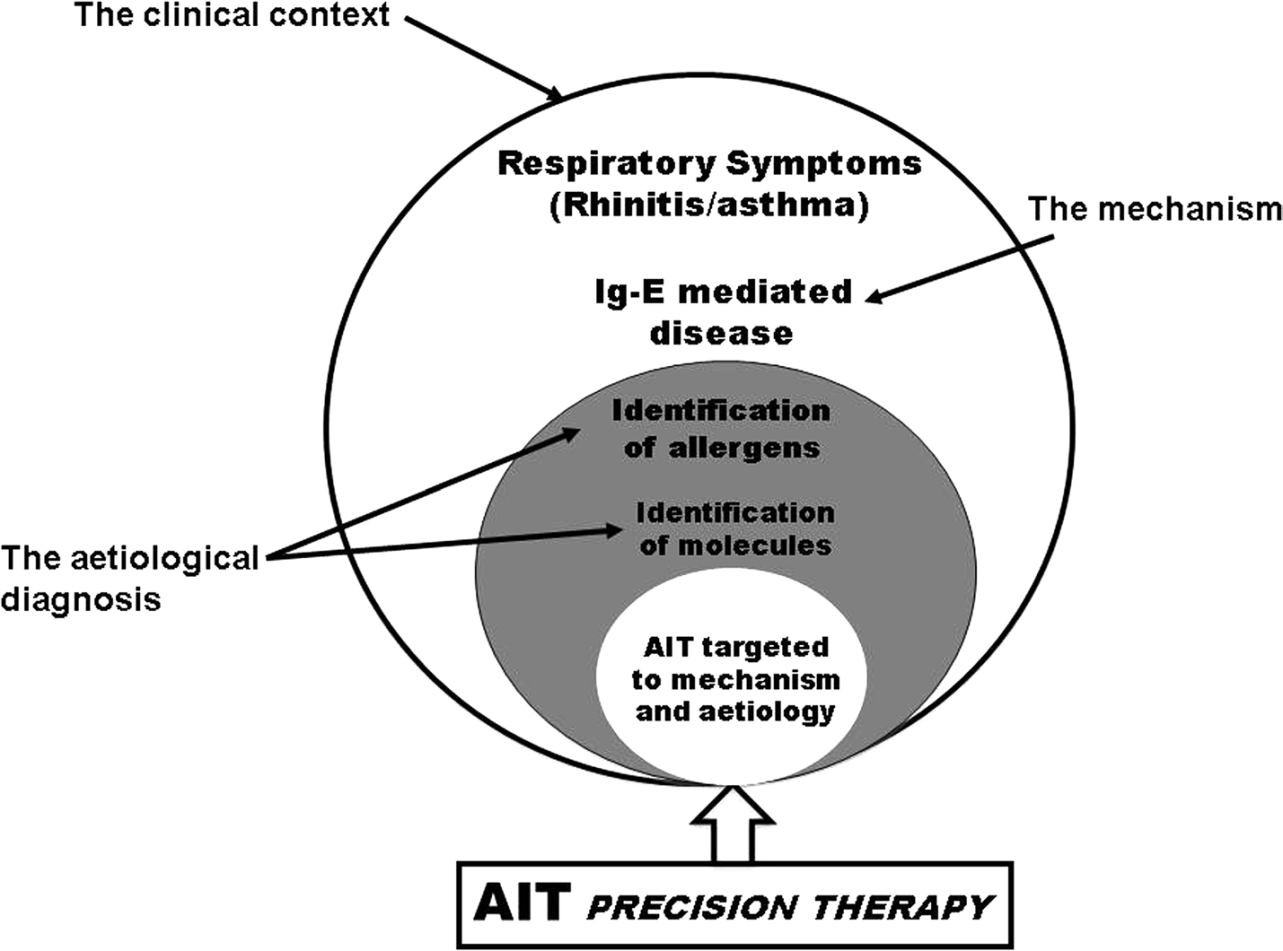
The role of allergen immunotherapy (AIT) as a personalized or precision medicine

Under a clinical point of view, AIT is well characterized by numerous clinical trials showing the effectiveness of the treatment [19–21], and this is even more apparent for sublingual immunotherapy (SLIT), for which large trials involving hundreds of patients are nowadays available [21]. According to this, we can perform an optimized prescription (Fig. 2) [17].

Allergen immunotherapy has a unique immunological rationale, tailored to the specific IgE spectrum of each individual. In addition, AIT can modify the immune response against the allergen for which the treatment is designed, thus allowing to modify the natural history of the disease itself [22], and this is a unique feature in treating allergies. In addition, the AIT long term effect remains a unique feature of great impact in the pharmaco-economic evaluation, since no other allergy treatment has this specific characteristic [23].

Nonetheless, we still need biomarkers which could be predictive of the expected efficacy and, consequently, the identification of the eligible patients, with a direct economic implication. We, would highlight other critical issues for a correct and effective AIT. Certainly we would need a more spread knowledge on molecular allergy, to be ourselves more adherent to the definition of personalized medicine. Moreover, a clear characterization and definition of commercial products for vaccination is also urgently needed. A precision medicine requires precision approaches, whereas nowadays, for many commercial products the characterization remains poor, and in some cases an experimental proof of efficacy is lacking [24]. This is the reason why, regulatory authorities are strenuously trying to provide clear rules for the marketing, and for AIT products commercial authorizations [25, 26].

## Conclusion

According to the current knowledge of mechanistic aspects, to the detailed identification of aetiological agents, and the not negligible longstanding experience, AIT, in the context of the other available therapies for respiratory allergy, is the most “personalized” treatment [27] (Fig. 2). Possibly, in the past, the concept of AIT as Precision Treatment was not properly considered or emphasized, but AIT was and still is upfront in this context. We are aware that more precise information and markers will be provided by systems medicine [28] and networks projects [29, 30]: these will further improve AIT indication, patient selection for clinical trials, prescription and, consequently, effectiveness and cost/effectiveness.

**Fig. 3:**
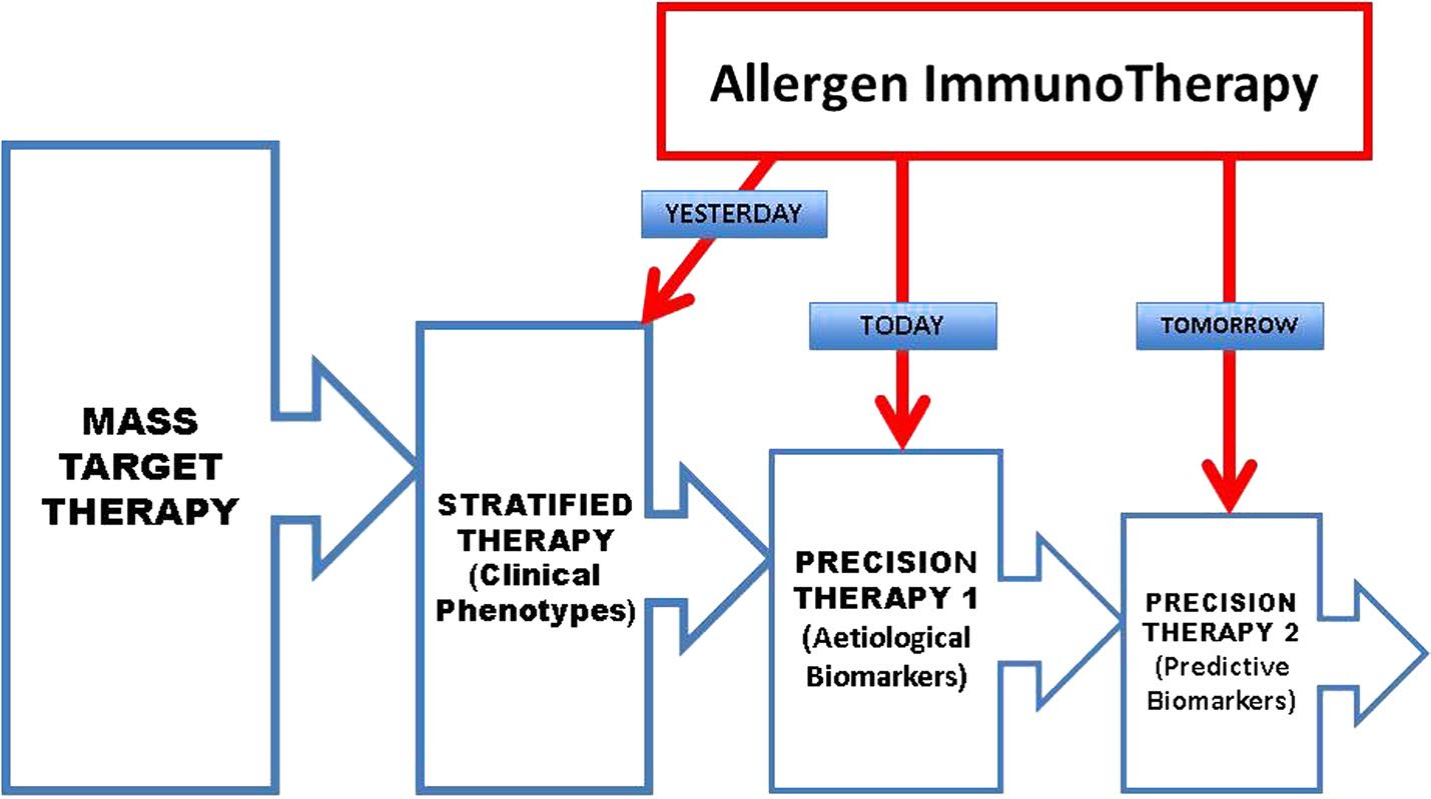
Allergen immunotherapy (AIT): the ideal pathway towards precision medicine

The area of medical technology is evolving rapidly and monitoring data will be critical to many aspects of developing AIT as precision medicine: finding patients with the required biomarkers (Fig. 3) for trials, monitoring efficacy and safety of targeted therapies will be also needed for reaching a justified sustainability. Finally, also partnerships between allergy scientific community, manufacturers and all other stakeholders in the healthcare system should be promoted and encouraged to achieve significant changes in medicine reimbursement.
